# Rituximab/bendamustine/cytarabine for transplant‐eligible patients with mantle cell lymphoma: A retrospective study

**DOI:** 10.1002/cam4.6114

**Published:** 2023-05-18

**Authors:** Yuki Shimizu, Daisuke Kaji, Otoya Watanabe, Kyosuke Yamaguchi, Kosei Kageyama, Yuki Taya, Aya Nishida, Kazuya Ishiwata, Shinsuke Takagi, Hisashi Yamamoto, Yuki‐Asano Mori, Atsushi Wake, Naoyuki Uchida, Shuichi Taniguchi, Go Yamamoto

**Affiliations:** ^1^ Department of Hematology Toranomon Hospital Tokyo Japan; ^2^ Department of Hematology Toranomon Hospital Kajigaya Japan

**Keywords:** effective and safe, induction therapy, mantle cell lymphoma, rituximab/bendamustine/cytarabine, transplant eligibility

## Abstract

**Background:**

Mantle cell lymphoma is considered an aggressive B‐cell lymphoma. The optimal induction regimen remains controversial as no randomized controlled trial has compared the efficacy of different induction therapies.

**Method:**

Herein, we performed a retrospective analysis of the clinical characteristics of 10 patients who received induction treatment consisting of rituximab, cyclophosphamide, doxorubicin, vincristine, and prednisone (R‐CHOP) and rituximab, bendamustine, and cytarabine (R‐BAC) at Toranomon Hospital between November 2016 and February 2022.

**Result:**

Although one patient discontinued R‐BAC therapy due to a rash, the other nine completed the scheduled chemotherapy. All patients achieved complete response, underwent high‐dose chemotherapy and autologous stem cell transplantation, and maintained complete remission with a median follow‐up of 15 months. Hematological adverse events (AEs) occurred in all patients; however, none developed documented infection. There were also no fatal non‐hematological AEs specific to R‐BAC.

**Conclusion:**

R‐CHOP/R‐BAC may be a good induction therapy for transplant‐eligible patients with mantle cell lymphoma.

## 1 INTRODUCTION

Mantle cell lymphoma (MCL), a B‐cell lymphoma that is aggressive in most cases, accounts for less than 10% of non‐Hodgkin lymphoma cases.^1^ Several studies have demonstrated the efficacy of upfront high‐dose chemotherapy with autologous stem cell transplantation (HDC/ASCT)^2^, ^3^, ^4^, ^5^; however, the optimal induction therapy before HDC/ASCT remains controversial as no randomized controlled trials have been performed that compare the different regimens.

Induction with the rituximab, cyclophosphamide, doxorubicin, vincristine, and prednisone (R‐CHOP) regimen does not sufficiently improve long‐term prognosis.^6^ Intensive induction therapy including high‐dose cytarabine (HDAraC) is effective.^5^, ^7^, ^8^, ^9^, ^10^ However, an increase in severe infection or renal dysfunction can occur following treatment with rituximab, hyperfractionated cyclophosphamide, vincristine, doxorubicin, and dexamethasone alternating with HDAraC and methotrexate (R‐hyperCVAD/MA) or with rituximab, dexamethasone, cytarabine, and cisplatin alternating with CHOP (R‐DHAP/CHOP). These adverse events (AEs) are reversible in most cases; nonetheless, the risk of a decreased response rate and relapse due to reduced chemotherapy doses is a concern.

Bendamustine‐containing regimens are effective induction therapies as well. Rituximab plus bendamustine and cytarabine (R‐BAC) has a high response rate with few severe complications in ASCT‐ineligible patients.^11^, ^12^ Armand et al. showed the efficacy of three cycles of rituximab and bendamustine followed by three cycles of rituximab and HDAraC (RB/RC) for ASCT‐eligible patients.^9^, ^10^ R‐BAC contains less cytarabine than RB/RC; however, as therapeutic synergy between bendamustine and cytarabine in MCL cells has been shown in vitro,^13^ R‐BAC may be effective as first‐line therapy for ASCT‐eligible patients.

Herein, we investigate the efficacy and safety of R‐CHOP/R‐BAC as induction therapy for ASCT‐eligible patients with MCL.

## 2 METHODS

### 2.1 Study design

In this single‐center retrospective study, an expert hematopathologist made a histological diagnosis of MCL according to the World Health Organization classification.^14^ Patients who had not been treated previously and had undergone HDC/ASCT at Toranomon Hospital between November 2016 and February 2022 were included.

### 2.2 Treatment

Induction therapy consisted of three sequential cycles of R‐CHOP (rituximab 375 mg/m^2^ [Day 1], cyclophosphamide 750 mg/m^2^ [Day 1], doxorubicin 50 mg/m^2^ [Day 1], vincristine 1.4 mg/m^2^ [Day 1], and prednisolone 60 mg/m^2^ [Days 1–5]), and three sequential cycles of R‐BAC (rituximab 375 mg/m^2^, bendamustine 70 mg/m^2^ [Days 1–2], and cytarabine 500 mg/m^2^ [Days 1–3]). In all cases, acyclovir and sulfamethoxazole/trimethoprim were used as prophylactic antibiotics during induction therapy. All patients underwent baseline computed tomography or ^18^F‐fluorodeoxyglucose–positron emission tomography (FDG‐PET) and bone marrow biopsy at diagnosis. Patients underwent peripheral blood stem cell harvesting (PBSCH) after two or three cycles of R‐BAC. The response was evaluated using FDG‐PET before HDC/ASCT, and bone marrow examination was performed in patients with bone marrow involvement at diagnosis. R‐MEAM (rituximab 375 mg/m^2^, ranimustine 300 mg/m^2^ [Day −6], etoposide 100 mg/m^2^ × 2 [Days −5 to −2], cytarabine 2 g/m^2^ [Days −3 to −2], and melphalan 140 mg/m^2^ [Day −1]) was used as a conditioning regimen before ASCT. R‐MEAM was shown to be effective in retrospective studies and is often used as a conditioning regimen in Japan because carmustine (used in R‐BEAM) is not available.^15^

### 2.3 Statistical analysis

We did not perform a sample size calculation and power analysis, although the sample size was small. Overall survival was calculated from the date of diagnosis of MCL until death from any cause, and progression‐free survival (PFS) was calculated from the date of diagnosis of MCL until disease progression or death using the Kaplan–Meier method.

AE severity was graded according to the Common Terminology Criteria for Adverse Events (CTCAE) version 5.^16^

## 3 RESULTS

### 3.1 Patients

Totally, 12 transplant‐eligible patients with MCL were treated at our hospital from November 2016 to February 2022. Among them, two patients were excluded; one patient because of concomitant diffuse large B‐cell lymphoma in the central nervous system and MCL in the rectum and bone marrow, and one patient who received six cycles of R‐CHOP therapy due to hypotension caused by the administration of bendamustine. Therefore, 10 patients were treated with R‐CHOP/R‐BAC as induction therapy. The patients' characteristics are summarized in Table 1. The median age at diagnosis of MCL was 50 years (range, 43–65 years), and all patients had an Eastern Cooperative Oncology Group performance status of 0 or 1 and were eligible for HDC/ASCT. The median MCL international prognostic index was 5.79 (range, 5.16–7.12). One patient had a blastoid variant but did not have symptoms indicating central nervous system involvement; therefore, we did not perform central nervous system prophylaxis. All patients achieved complete response (CR) and underwent ASCT conditioned with R‐MEAM.

**TABLE 1:** Patient characteristics (*n* = 10).

Median age at diagnosis, years (range)	50 (43–65)
Sex	
Male	7
Female	3
Stage at diagnosis	
III	1
IV	9
ECOG PS	
0	7
1	3
MIPI score	
Low	4
Intermediate	4
High	2
IPI	
Low	4
Low‐intermediate	4
High‐intermediate	2
Ki‐67 > 30%	2
Morphological subtypes	
Blastoid	1
Others	9

Abbreviations: ASCT, autologous stem cell transplantation; CR, complete remission; ECOG PS, Eastern Cooperative Oncology Group Performance Status; IPI, international prognostic index; MIPI, mantle cell lymphoma international prognostic index.

### 3.2 PBSCH

G‐CSF was used to mobilize stem cells in all patients. In addition to G‐CSF, plerixafor was used in seven patients, and a sufficient number of CD34^+^ cells was harvested in one series of mobilization in all patients except one. Plerixafor had not been used for this patient, but an additional PBSCH after the third cycle of R‐BAC yielded a collection of 1.37 × 10^6^ CD34^+^ cells/kg. The median number of days required for PBSCH was 1 (range, 1–4).

### 3.3 Engraftment

All patients succeeded in engraftment, and the median times for neutrophil, reticulocyte, and platelet engraftments were 10.5 days (range, 9–12), 14 days (range, 12–28), and 21 days (range, 12–92), respectively.

### 3.4 Efficacy

We did not perform an interim evaluation between R‐CHOP and R‐BAC, but we evaluated the efficacy after completion of induction therapy. All patients were assessed as having achieved complete metabolic response using the Deauville five‐point scale before ASCT, and bone marrow examinations were negative in all eight patients who had bone marrow involvement at diagnosis. Six patients received rituximab maintenance therapy after HDC/ASCT. The median follow‐up period after ASCT was 15 months, and all patients maintained CR.

### 3.5 Toxicity

One patient discontinued R‐BAC therapy after two cycles owing to a Grade 2 skin rash, but the rash subsequently improved. The other nine patients completed the scheduled R‐CHOP/R‐BAC therapy. No treatment‐related deaths occurred during this period. Grade 3 or 4 neutropenia occurred in all patients, and febrile neutropenia (FN) occurred in two (20%); however, there was no lethal infection. Regarding non‐hematological AEs, Grade 3 ejection fraction reduction occurred in one patient; no other Grade 3 or higher AEs were observed (Table 2).

**TABLE 2:** Adverse events of induction therapy and clinical data on ASCT.

AEs	G3	G4
Neutropenia	4	6
Leukopenia	1	9
Thrombopenia	2	7
Anemia	6	0
FN	2	
EF decreased	1	0
PBSCH/PBSCT		
Mobilization		
G‐CSF only	3	
G‐CSF + plerixafor	7	
Median of CD34^+^ cells (×10^6^/kg)	6.04 (1.37–42.34)	
Median day of engraftment		
Neutrophils (>500/μL)	10.5 (9–12)	
Thrombocytes (>50,000/μL)	21 (12–92)	
Reticulocytes (>1‰)	14 (12–28)	

*Note*: Data refer to patients with at least 1 day of Grade 3 or 4 events throughout all cycles.

Abbreviations: AEs, adverse events; EF, ejection fraction; FN, febrile neutropenia; G‐CSF, granulocyte colony‐stimulating factor; PBSCH, peripheral blood stem cell harvest; PBSCT, peripheral blood stem cell transplantation.

## 4 DISCUSSION

To the best of our knowledge, this is the first report that demonstrates the use of R‐CHOP/R‐BAC therapy as induction therapy prior to HDC/ASCT. Visco et al. demonstrated in a Phase 2 study that induction therapy with R‐BAC for untreated older patients with MCL had a 100% overall response rate and 95% 2‐year PFS rate.^11^ Based on these findings, we treated newly diagnosed ASCT‐eligible patients with MCL with R‐CHOP/R‐BAC. All patients achieved CR and maintained it during the follow‐up period.

The frequency of hematological toxicity with R‐CHOP/R‐BAC was higher than that with HDAraC‐containing regimens. Grade 3 or 4 neutropenia was particularly observed in all patients treated with R‐CHOP/R‐BAC, but the FN rate was comparable with that in other regimens, and no severe infection was documented. During R‐CHOP/R‐DHAP and RB/RC, Grade 3 or 4 neutropenia and FN were observed in 74% and 17%, and 83% and 15% of patients, respectively, and Grade 3 or higher infection occurred.^10^, ^17^ This may be because the time to hematopoietic recovery is relatively short after R‐BAC. Additionally, the percentage of patients with successful collection of 2 × 10^6^ CD34^+^ cells/kg was 66% after R‐DHAP/CHOP^17^ and 98% after RB/RC,^10^ and the CD34^+^ cell collection rate after R‐BAC was comparable to that of these regimens.

Only one patient developed Grade 3 or higher non‐hematological toxicity, which resulted in a reduction of the ejection fraction. We concluded that the cardiotoxicity was caused by R‐CHOP because it occurred prior to starting R‐BAC, and the doxorubicin contained in R‐CHOP is known to cause cardiotoxicity in a dose‐dependent manner.^18^, ^19^ One patient discontinued R‐BAC after two cycles due to a Grade 2 skin rash, which was diagnosed as eosinophilic pustulosis by a dermatologist. A Grade 2 or lower skin rash following treatment with a bendamustine‐containing regimen has been previously reported, but it did not require discontinuance of the regimen.^10^, ^11^, ^12^ In our study, the skin rash was Grade 2 and improved with topical steroid administration; nonetheless, we changed the regimen because it was intractable. Common non‐hematological AEs of R‐BAC have been previously reported^11^; our study included few severe non‐hematological AEs (Table S1).

Induction therapy with BR alone for ASCT‐eligible patients with newly diagnosed MCL demonstrated an overall response rate of 89% (CR rate 61%),^20^ and R‐BAC improved the 2‐year PFS compared with RB in ASCT‐ineligible patients (87% vs. 64% [*p* = 0.001]), while the toxicity was tolerable.^21^ The addition of cytarabine may improve the survival of ASCT‐eligible patients as well, and remission induction therapy with R‐BAC alone may be worth considering.

The limitation of this study is the small number of cases and short observation period. A study with a larger cohort is needed to determine whether R‐CHOP/R‐BAC improves the long‐term survival of patients with MCL. Testing the efficacy of induction therapy with R‐BAC alone, followed by HDC/ASCT, may be beneficial. Additionally, screening for genetic mutations, such as *MYC* and *TP53*, was not performed at diagnosis at our hospital. ASCT is less beneficial for patients with a *TP53* mutation, and the impact of this genetic characteristic was not considered in this study.

## AUTHOR CONTRIBUTIONS

**Yuki Shimizu:** Writing – original draft (equal). **Daisuke Kaji:** Writing – review and editing (lead). **Otoya Watanabe:** Writing – review and editing (supporting). **Kyosuke Yamaguchi:** Writing – review and editing (supporting). **Kosei Kageyama:** Writing – review and editing (supporting). **Yuki Taya:** Writing – review and editing (supporting). **Aya Nishida:** Writing – review and editing (supporting). **Kazuya Ishiwata:** Writing – review and editing (supporting). **Shinsuke Takagi:** Writing – review and editing (supporting). **Hisashi Yamamoto:** Writing – review and editing (supporting). **Yuki Asano Mori:** Writing – review and editing (supporting). **Atsushi Wake:** Writing – review and editing (supporting). **Naoyuki Uchida:** Writing – review and editing (supporting). **Shuichi Taniguchi:** Writing – review and editing (supporting). **Go Yamamoto:** Writing – review and editing (supporting).

## FUNDING INFORMATION

This research received no specific grant from funding agencies in the public, commercial, or not‐for‐profit sectors.

## CONFLICT OF INTEREST STATEMENT

All authors have no conflict of interest.

## ETHICS STATEMENT

This study complied with the provisions of the Declaration of Helsinki and was approved by the Institutional Review Board of Toranomon Hospital, Tokyo, Japan (approval no. 2115). The requirement for informed consent was waived because of the retrospective nature of the study. As this study was not registered, no registration number is available. This study was not an animal study.

## Supporting information


Table S1.

## Data Availability

The data that support the findings of this study are available from the corresponding author upon reasonable request.
