# Supplementary material for: Rituximab/bendamustine/cytarabine for transplant‐eligible patients with mantle cell lymphoma: A retrospective study
Source: Cancer Med. 2023 May 18;12(11):12548–52. doi: 10.1002/cam4.6114 (PMC10278520; doi:10.1002/cam4.6114)
Supplement: Supplementary file 1 — Table S1. [file CAM4-12-12548-s001.docx]

**Supplemental Table 1.** Comparison of the efficacy and adverse events of induction therapy with previous studies

AE, adverse event; AKI, acute kidney injury; AST/ALT, aspartate aminotransferase/alanine aminotransferase; BAC, bendamustine and cytarabine; CHASER, rituximab, cyclophosphamide, cytarabine, dexamethasone, and etoposide; CHOP, cyclophosphamide, doxorubicin, vincristine, and prednisone; CR, complete remission; DHAP, dexamethasone, cytarabine, and cisplatin; FN, febrile neutropenia; G3, grade 3; G4, grade 4; HDAraC, high-dose cytarabine; HDC/ASCT, high-dose chemotherapy and autologous stem cell transplantation; Hyper CVAD, hyperfractionated cyclophosphamide, vincristine, doxorubicin, and dexamethasone; MA, high-dose cytarabine and methotrexate; PR, partial response; R, rituximab; RB/RC, rituximab and bendamustine followed by rituximab and high-dose cytarabine; TRM, treatment-related mortality.

| Regimen | HDC/ ASCT | Patients (n) | Age  (median; years) | Hematological AE  (G3 or G4) | | Non-hematological AE  (G3 or G4) | | Discontinued due to toxicity | TRM  (n) | Response | Reference |
| --- | --- | --- | --- | --- | --- | --- | --- | --- | --- | --- | --- |
| R-DHAP/CHOP | Eligible | 248 | 56 (50–60) | Neutropenia  Thrombopenia  Anemia  FN | 74%  73%  29%  17% | Cre elevation  Infection  Nausea  Vomiting | 1%  9%  4%  3% | N/A | N/A | CR 55%  CRu 38% | 17 |
| R-Hyper CVAD/MA | Eligible | 63 | 57 (29–66) | Neutropenia  Thrombopenia  Anemia  FN | 94%  69%  44%  6% | Infection cardiac arrhythmia paralytic ileum thromboembolic event acute pancreatitis skin reaction peripheral neuropathy | 50%  6%  1%  3%  1%  1%  1% | 17% | 4% | CR 72%  PR 11% | 8 |
| R-MaxiCHOP/HDAraC | Eligible | 176 | 56 (32–65) | FN | 12% | Infection | 4% | 2.8% | 5% | CR 45.6%  CRu 8.8% | 3 |
| R-High-CHOP/CHASER | Eligible | 45 | 59 (38–65) | Neutropenia  Thrombopenia  Anemia | 100%  98%  91% | AST elevation  ALT elevation  Hypokalemia | 7%  11%  22% | 6.7% | 2% | CR 82.2% | 5 |
| RB/RC | Eligible | 88 | 58 (30–72) | Neutropenia  Lymphopenia  Thrombopenia  Anemia  FN | 83%  88%  85%  44%  15% | Enterocolitis  Pneumonia  Sepsis  Hyperglycemia  Hyperuricemia  TTP | 2%  2%  2%  2% 2%  2% | 2% | 0% | CR 90%  PR 7% | 10 |
| R-CHOP/BAC | Eligible | 10 | 50 (43–65) | Neutropenia  Lymphopenia  Thrombopenia  Anemia  FN | 100%  100%  90%  60%  20% | Ejection fraction decreased | 10% | 10% | 0% | CR 100% | Toranomon hospital |
